# Standing balance of professional ballet dancers and non-dancers under different conditions

**DOI:** 10.1371/journal.pone.0224145

**Published:** 2019-10-22

**Authors:** Miroslav Janura, Markéta Procházková, Zdeněk Svoboda, Lucia Bizovská, Soňa Jandová, Petr Konečný

**Affiliations:** 1 Department of Natural Sciences in Kinanthropology, Faculty of Physical Culture, Palacký University Olomouc, Olomouc, Czech Republic; 2 Department of Applied Mechanics, Faculty of Mechanical Engineering, Technical University of Liberec, Liberec, Czech Republic; 3 Department of Physiotherapy, Faculty of Health Sciences, Palacký University Olomouc, Olomouc, Czech Republic; Universitat Konstanz, GERMANY

## Abstract

Ballet training has been reported to positively influence balance ability. It is not entirely clear how improved balance ability manifests under standing conditions with different demands on postural control. The aim of the study was to compare balance of ballet dancers and non-dancers in a unipedal stance under different conditions. Twenty-five professional ballet dancers and twenty-five controls completed four unipedal standing balance tests: firm surface with eyes open and closed; foam mat surface with eyes open; and firm surface with eyes open immediately after performing ten 360° whole-body turns. The centre of pressure (COP) data were obtained with a force platform and the direction-specific standard deviations, velocities, and sample entropy of the COP displacement were computed. A three-way analysis of variance was used to compare groups, genders, and conditions. For standing immediately after performing ten turns, the postural sway parameters were significantly larger in the control group compared to the ballet dancers in both men and women. In this stance condition the values of postural sway and COP velocities in the control group were larger in the men compared to the women. For both genders in the control group all postural sway and COP velocity parameters were larger in standing with eyes closed and standing after performing 10 turns compared to standing with eyes open on both firm and foam surface. In the ballet dancers all COP velocity parameters were larger in standing with eyes closed compared to all other conditions. The results from the present study indicate that professional ballet dancers do not have a better general balance ability than untrained subjects.

## Introduction

Ballet as a unique combination of art and sport places high demands on the dancer’s musculoskeletal system and affects the motor behaviour of the dancers. Dance performance is a complex act with a large number of elements–strength, balance, flexibility etc. [1].

For ballet dance are typical specific movements which require precise spatial and temporal coordination of multi-joint limb movement with postural control [2]. A high level of postural control is important for ballet dancers to achieve an optimum aesthetic level of dance performance [3], it also may reduce the risk for musculoskeletal injuries [2]. Good level of static and dynamic balance, most often in a unipedal stance, as well as the ability to turn efficiently are some of the most important requirements put on the ballet dancers [3].

Ballet dancers use specific equilibrium exercises [4]. It can be expected, that dancers will have a highly advanced sense of awareness for the placement and motion of their bodies [5]. External conditions of lighting and support surfaces in ballet training vary, so dancers may be better able to use information obtained from the somatosensory, and vestibular systems [6]. It could be assumed, that the impact of ballet training would have a positive effect on the postural stability in standing. However, Giboin et al. [7] reported that balance training resulted in highly task-specific effects and it did not improve general balance ability. Similarly, Hugel et al. [8] suggested that the balance abilities developed through the ballet dancers’ specialized training may not transfer to the less-challenging balance conditions that are more typical of the activities of daily living.

This may also be the reason for equivocal results when comparing postural control of ballet dancers and untrained subjects. Golomer et al. [9] found that professional ballet dancers were less dependent on vision for postural control and for perception than non-dancers. Conversely, de Mello et al. [10] concluded that the visual dependency of professional ballet dancers for balance adjustment was greater and the influence of the supporting base on postural sway in professional balance dancers was reduced.

Michalska et al. [11] reported that professional dancers had larger postural sway characteristics in comparison to the non-dancers while performing simple motor tasks. Visual information played an important role in the process of maintaining a stable position of the dancer’s body [11]. Simmons [12] found that dancers demonstrated better balance due to more consistent neuromuscular responses, higher proprioceptive sensitivity and they were also more consistent in muscle activation. Golomer et al. [9] suggested that one of the reasons why professional dancers were less dependent on vision for dynamic postural control was that dance training presumably shifts the sensorimotor dominance from vision to proprioception.

To obtain more information about balance in professional ballet dancers, it can be useful to evaluate not only the basic parameters of the COP movement, but also to assess the temporal structure of the COP signal using sample entropy. Irregularity and, thus, high entropy (automatic control processes) during postural task can be interpreted as a sign of a healthy, vigilant system [13].

It is still unclear whether postural control in professional ballet dancers is different than in untrained subjects in standing balance under different sensory inputs. Therefore, the aim of this study was to assess whether there are differences in postural control between ballet dancers and non-dancers and if these differences are associated with the demands of a postural task. To compare the standing balance between both groups the unipedal standing was chosen, which reduces the quantity of useful and accurate somatosensory information available to the postural control system [14]. We hypothesized that increase in demands of the task results in increase in difference between dancers and non-dancers, with dancers performing better.

## Materials and methods

### Participants

A group of twenty-five professional ballet dancers (twelve women and thirteen men) employed at the National Theatre in Brno and the Moravian Theatre in Olomouc participated in the study. None of them were injured at that time and all were performing on a regular basis. All the dancers had at least ten years of ballet dancing experience. They practiced five to six days per week, three to eight hours per day. The control group was composed of twenty-five age-matched healthy adults (fourteen women and eleven men), who did not compete in sport at elite level, who were free from injury (no musculoskeletal injuries or neurological conditions) and who had no experience with dance training or any other form of specialized balance training. The study group demographics are presented in Table 1. All participants were informed about the purpose of the study and signed a written consent. The study was approved by the Ethics Committee of the Faculty of Physical Culture, Palacký University Olomouc and the procedures presented were in accordance with the ethical standards for human experimentation as stated in the Helsinki Declaration.

**Table 1:** Physical characteristics of participants (Mean ± *SD*).

	Women			Men		
	Ballet	Controls	*p*	Ballet	Controls	*p*
Age (years)	25.6 ± 3.8	24.7 ± 2.6	0.716	23.4 ± 4.0	23.6 ± 1.6	0.157
Mass (kg)	49.5 ± 3.7	65.6 ± 9.2	**< 0.001**	67.7 ± 6.5	71.1 ± 7.8	0.268
Height (cm)	164.1 ± 4.4	169.6 ± 6.2	**0.023**	177.9 ± 4.3	177.8 ± 5.6	0.806
BMI (kg/m^2^)	18.4 ± 0.9	22.8 ± 2.9	**< 0.001**	21.4 ± 1.8	22.5 ± 2.0	0.148

*p*-values < 0.05 are in bold.

### Postural stability

Postural stability data were obtained with a Kistler 9286AA force platform (Kistler Instrumente AG, Winterthur, Switzerland; 600 x 400 x 35 mm, sampling frequency of 200 Hz). Postural stability was tested in the unipedal stance (for both legs) in four different conditions:

1. standing on a firm surface with eyes open,
2. standing on a firm surface with eyes closed,
3. standing on a foam mat surface (Airex Balance Pad, Airex AG, Sins, Switzerland) with eyes open,
4. standing on a firm surface with eyes open after performing ten 360° non-travelling whole-body turns.

The participants were instructed to stand barefoot in an upright posture on the force platform, with their arms relaxed at their sides, while looking at the visual target placed at eye level on the wall in the distance of 2.5 m. Participants were instructed to stand as still as possible for every trial. The whole-body turns were performed when standing on both legs at a self-selected speed with eyes open. The participants were instructed to take the unipedal stance immediately after finishing the turns, data recording started after five seconds of standing in the unipedal stance. Participants performed three trials for each condition, for a total of 24 trials. Each trial lasted 30 seconds. A rest period of 60 to 90 seconds was given between the trials to prevent fatigue. The order of the tests was randomized for each participant.

The data obtained from the force plate were filtered by a 4th-order low-pass bidirectional Butterworth filter with a cut-off frequency of 7 Hz. A custom MATLAB (version 2018a, MathWorks, Inc., Natick, MA, USA) script was used to compute the standard deviation of the displacement of the COP in the medial-lateral (ML) and antero-posterior (AP) directions (Sway X and Sway Y, respectively), the mean velocities in the ML (Vx) and AP (Vy) directions, and the mean total velocity (V) of the COP translation. Moreover, sample entropy was computed from COP coordinates for each direction (SEx, SEy). Sample entropy is computed as the negative of natural algorithm of a conditional probability that two similar vectors with the length of *m* consecutive data points will remain similar with tolerance *r* even after adding one more data point [15]. The computation was performed for *m* = 2, similarity criterion *r* = 0.15 [15] using an algorithm available on Physionet [16–18].

### Statistical analyses

Data analyses were performed using the STATISTICA (version 12.0, StatSoft, Inc., Tulsa, OK, USA) software. The mean of the three trials was used in the analysis. No statistical differences were found between dominant and non-dominant leg in a preliminary analysis, therefore, the averaged data of both legs were used further. A three-way analysis of variance (ANOVA) with 2x2x4 design was used to compare groups (ballet, control), gender (men, women) and conditions (1–4). The effect was considered significant for *p* < 0.05. Partial η^2^ was used as an effect size indicator. Furthermore, Tukey post-hoc test was implemented for pairwise comparisons.

## Results

### Physical characteristics of participants

The physical characteristics of all participants are shown in Table 1. The women ballet dancer group had significantly lower body mass (*p* < 0.001), body height (*p* = 0.023), and body mass index (*p* < 0.001) than the control group.

### Postural control

#### Group*condition interaction

Group*condition interaction was significant for postural sways, COP velocity parameters and sample entropy in the antero-posterior direction (Sway X: *F* = 21.645, *p <* 0.001, η^2^ = 0.261; Sway Y: *F* = 22.996, *p <* 0.001, **η**^2^ = 0.273; Vx: *F* = 15.869, *p <* 0.001, **η**^2^ = 0.206; Vy: *F* = 25.120, *p <* 0.001, **η**^2^ = 0.291; V: *F* = 24.409, *p <* 0.001, **η**^2^ = 0.285; SEx: *F* = 1.947, *p* = 0.124, **η**^2^ = 0.031; SEy: *F* = 3.004, *p* = 0.032, **η**^2^ = 0.047).

#### The effect of group

Our results showed significant effect of group (ballet dancers, controls) on postural sway in the medial-lateral (*F* = 8.242, *p* = 0.005, **η**^2^ = 0.043) and antero-posterior directions (*F* = 8.687, *p* = 0.004, **η**^2^ = 0.045). In the COP velocity parameters, the effect of group was not significant. In the sample entropy parameters the effect of group was significant for both medial-lateral (*F* = 5.636, p = 0.019, **η**^2^ = 0.030) and antero-posterior directions (*F* = 21.354, *p* < 0.001, **η**^2^ = 0.104).

**Fig 1:**
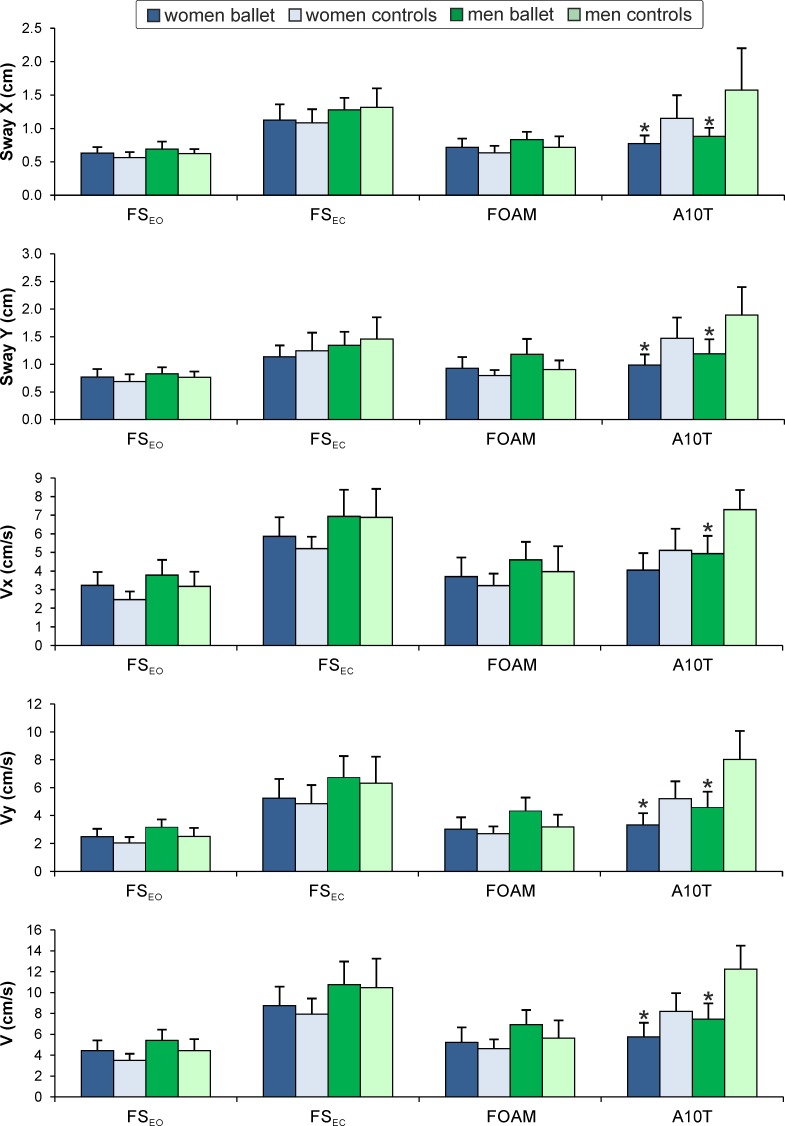
Mean (+ 1 *SD*) of the COP parameters during the four standing conditions. Sway X, Sway Y = standard deviation of the displacement of the COP in the medial-lateral, antero-posterior direction; Vx, Vy = mean velocity of the COP in the medial-lateral, antero-posterior direction; V = total velocity of the COP translation; FS_EO_ = firm surface, eyes open; FS_EC_ = firm surface, eyes closed; FOAM = foam surface (eyes open); A10T = firm surface, after performing 10 turns (eyes open). * indicates a statistically significant difference between the ballet dancers and the control group of the same gender.

Post hoc tests showed (Fig 1), that for standing immediately after performing 10 turns, the postural sway parameters were significantly larger in the control group compared to the ballet dancers in both men and women (Sway X: men *p <* 0.001, women *p* = 0.001; Sway Y: men and women *p* < 0.001). We did not find any significant difference between the groups in the sample entropy parameters, except for SEy in the men when standing with eyes closed (*p* = 0.041; Table 2).

**Table 2:** Sample entropy values (Mean ± *SD*).

	Women			Men		
	Ballet	Controls	*p*	Ballet	Controls	*p*
SEx						
FS_EO_	0.131 ± 0.032	0.111 ± 0.024	0.954	0.145 ± 0.031	0.137 ± 0.042	0.999
FS_EC_	0.149 ± 0.028	0.130 ± 0.026	0.961	0.148 ± 0.028	0.147 ± 0.031	0.999
FOAM	0.131 ± 0.024	0.139 ± 0.030	0.999	0.147 ± 0.027	0.151 ± 0.045	0.999
A10T	0.139 ± 0.027	0.115 ± 0.027	0.806	0.152 ± 0.021	0.130 ± 0.041	0.941
SEy						
FS_EO_	0.074 ± 0.020	0.061 ± 0.016	0.956	0.092 ± 0.024	0.074 ± 0.023	0.753
FS_EC_	0.112 ± 0.028	0.090 ± 0.013	0.276	0.128 ± 0.025	0.098 ± 0.016	**0.041**
FOAM	0.074 ± 0.017	0.079 ± 0.018	0.999	0.092 ± 0.022	0.083 ± 0.019	0.999
A10T	0.071 ± 0.019	0.063 ± 0.018	0.999	0.088 ± 0.027	0.075 ± 0.017	0.977

SEx = sample entropy in the medial-lateral direction; SEy = sample entropy in the antero-posterior direction; FS_EO_ = firm surface, eyes open; FS_EC_ = firm surface, eyes closed; FOAM = foam surface (eyes open); A10T = firm surface, after performing 10 turns (eyes open). *p*-values < 0.05 are in bold.

#### The effect of gender

A significant effect of gender was confirmed for all variables (Sway X: *F* = 23.411, *p <* 0.001, **η**^2^ = 0.113; Sway Y: *F* = 27.715, *p <* 0.001, **η**^2^ = 0.131; Vx: *F* = 59.701, *p <* 0.001, **η**^2^ = 0.245; Vy: *F* = 60.315, *p <* 0.001, **η**^2^ = 0.247; V: *F* = 68.693, *p <* 0.001, **η**^2^ = 0.272; SEx: *F* = 10.528, p = 0.001, **η**^2^ = 0.054; SEy: *F* = 21.459, *p <* 0.001, **η**^2^ = 0.104).

Post hoc analysis showed larger values of postural sway and COP velocities in the men compared to the women only for the controls, for all parameters in standing immediately after performing 10 turns (Sway X, Vx, Vy, V: *p <* 0.001; Sway Y: *p* = 0.005) and for the COP velocity in the medial-lateral direction (Vx: *p* = 0.003) in standing with eyes closed. For the sample entropy parameters we did not find any significant difference in both ballet dancers and control groups.

#### The effect of condition

The effect of condition was significant for all variables with exception of sample entropy in the medial-lateral direction (Sway X: *F* = 75.855, *p <* 0.001, **η**^2^ = 0.553; Sway Y: *F* = 64.384, *p <* 0.001, **η**^2^ = 0.512; Vx: *F* = 96.136, *p <* 0.001, **η**^2^ = 0.611; Vy: *F* = 92.550, *p <* 0.001, **η**^2^ = 0.601; V: *F* = 108.939, *p <* 0.001, **η**^2^ = 0.640; SEx: *F* = 1.997, *p* = 0.116, **η**^2^ = 0.032; SEy: *F* = 27.565, *p <* 0.001, **η**^2^ = 0.310).

Post hoc analysis showed for both genders significantly larger postural sway and COP velocity parameters in the control group in standing with eyes closed and standing after performing 10 turns compared to standing with eyes open on both firm and foam surfaces (*p <* 0.001). In the ballet dancers, we found significantly higher COP velocity parameters in standing with eyes closed compared to all other conditions (*p <* 0.001). Sway X was significantly larger in standing with eyes closed compared to standing with eyes open on both firm and foam surfaces (*p <* 0.001) and also compared to standing after performing 10 turns (only men, *p* = 0.002). For Sway Y we found a significantly larger value in standing with eyes closed compared to standing with eyes open on the firm surface (only men, *p <* 0.001).

For sample entropy, post hoc test showed significantly higher values only in the ballet dancers in the antero-posterior direction in standing with eyes closed compared to all other conditions (*p <* 0.002).

## Discussion

This study found that, for the unipedal stance, the sway and the velocity characterising the COP movement in the ballet dancers and the control group were generally comparable, with the exception of standing after performing 10 turns.

Comparable values of COP parameters could indicate a similar level of balance in ballet dancers and untrained subjects. However, balance assessment should not be done based only on the information about the amount of movement of the COP. It is important to focus also on the assessment of the temporal structure of the signal and the neuromuscular control strategy. This was found to be positively related to the degree of attention invested in postural control [19]. Roerdink et al. [20] state, that the automatic control processes increase the entropy of the signal while the volitional control decreases it. A more regular COP pattern indicates that the postural behaviour is more rigid [21].

In our study, the ballet dancers had significantly smaller postural sway and COP velocities compared to the controls only during standing after 10 turns. Because ballet dance training includes specific equilibrium exercises, which allow the dancers to perform balanced poses and hold their position for many seconds [22], it could be expected that the ballet dancers would exhibit better postural control in static conditions than non-dancers would. This was corroborated by previous studies [12, 23]. By contrast, Lin et al. [24], who assessed postural stability in the unipedal stance in different ballet postures in healthy non-dancers, healthy dancers, and dancers with ankle sprains, did not find any differences between the dancers and non-dancers during the static test performed both with the eyes open and closed. Similar results were also reported by Perrin et al. [25] who analysed postural balance control in static and dynamic conditions of high-level judoists, professional dancers, and the control group. Only the judoists were able to maintain a better balance control than the control group in all measured situations.

The results of our study also showed that the difference in the entropy values between ballet dancers and controls was not significant. Michalska et al. [11] and Stins et al. [19], who assessed sway patterns of ballet dancers in bipedal standing, presented increased values of sample entropy for ballet dancers, thus more automatized postural control. It seems that unipedal stance requires more attention (is less automatized) also in ballet dancers.

Another factor observed in this study was gender. Our results support the hypothesis that no gender differences would be found in the common balance tasks [26, 27]. On the other hand, in our study the men from the control group had a significantly larger postural sway and higher COP velocity in both directions after performing 10 turns compared to women from the same group. The reason for this is not clear and it seems that any explanation, perhaps except for the difference in body height, would be rather speculative.

Our findings further showed that differences between the ballet dancers and the control group are influenced by postural task. The effect of group*condition interaction was large for postural sway and COP velocities and small for sample entropy parameters. During standing with eyes closed significantly larger postural sway and higher COP velocities compared to all other conditions were found. Input from the visual system is fundamental in proactive (anticipatory) postural control [28]. The importance of visual input increases in situations where information from other sensory inputs is reduced [29, 30]. Such situations include also a unipedal stance used in our study.

The findings of Hugel et al. [8] demonstrate that the bipedal quiet stance is not representative of the conditions in which balance expertise comes into play. This was corroborated by Kiefer et al. [31] who compared proprioception awareness in dancers and non-dancers. They found that COP variability during quiet standing did not differ between both groups, in quiet stance ballet dancers did not benefit from their superior proprioceptive awareness. Common ballet training was not found to improve joint position sense at the ankle. Additional coordinative training is necessary to improve static balance of the ballet dancer [23]. This was the reason why, in the current study, postural stability was assessed using the unipedal stance, which is more demanding than the bipedal stance. The unipedal stance requires greater motor control than the bilateral stance because standing balance recovery movements at both the ankle and hip joints are needed. If the difficulty of balance task increases as encountered in the unipedal stance, the postural strategies used to recover balance could be reorganized [32]. Our findings showed that even during a unipedal stance with eyes open on both firm and foam surface the ballet dancers were not able to utilize their experience with balance training.

The postural control measurements obtained during standing after performing whole-body turns displayed a different tendency than did the other standing conditions. As the vestibular system plays an important role in balance, posture, and dynamic motion through space, the whole-body turns served in the present study as a specific vestibular manipulation. All observed parameters were significantly lower in the ballet dancers than in the control group for both men and women. These findings are in accordance with those by Hopper et al. [33].

One of the reasons of the differences found in our study may be that although pirouettes are a complex task, they are performed routinely by professional classical ballet dancers [34]. The ballet dancers’ ability to perform multiple pirouettes (the whole-body turns) with minimal sensation of vertigo could be result of effect of training on vestibular processing. According to Lin et al. [35] a smaller inclination angle of rotational axis is typical for experienced dancers which implies better biomechanical factors for maintaining postural stability. Pirouetting elicits the perception of rotatory self-motion (vertigo) as well as a reflexive vestibular-ocular reflex [36]. To be able to properly execute a pirouette, the ability to adapt motor behaviour based on imagery strategies is of major importance. It was concluded that professional dancers were able to compensate for vestibular and fatiguing perturbations due to a higher level of skill-specific motor training [33]. The non-dancers therefore lack the specific skills that the ballet dancers obtained during their ballet dance training.

When interpreting the results, balance should not be assessed as a general ability, it should be assessed as a task-specific skill [7, 37]. Casabona et al. [38] investigated the effect of foot configuration during bipedal stance on balance. They found that the differences between the professional ballet dancers and controls were only for stance with feet in extra rotation (an opening angle of 140°), thus in a stance familiar to the dancers. Improved balance resulting from the dance training was therefore related with a specific foot position and not with the level of stance difficulty. In the present study a unipedal stance under different sensorimotor conditions (open vs. closed eyes, firm vs. foam surface, vestibular manipulation) was used. The only statistically significant differences between the ballet dancers and controls were found for standing after performing 10 whole-body turns. While for the controls this condition seemed the most difficult, in the ballet dancers the values of COP sway and velocities were comparable to standing on the firm surface with eyes open and standing on the foam surface. This suggests that these differences can be explained by the task-specific skill of the ballet dancers they obtained by frequently performing various types of turns during dance training and performance.

In a group of ballet dancers also sample entropy in the antero-posterior direction was increased during this condition. This suggests lower attention during this task, probably due to reduction of information from vision. As ballet dancers use visual afference as the major input to achieve a better postural control, they can perform worse than untrained subjects in daily situations where this input is unavailable. [25].

Differences in sample entropy values during standing after performing whole-body turns between the ballet dancers and the controls were not significant in the women. We suppose that while in the ballet dancers the signal irregularity can be considered as a sign of automaticity, in the control group, comparable entropy values are a sign of postural instability. Kiefer et al. [39] stated that dancers were able to produce stable coordination patterns without rigidly constraining the coupling between movement system degrees of freedom. While adopting an automatic postural control, balance of non-dancers seemed to reach a plateau.

### Limitations of the current study

Among the limitations of the present study are the significantly lower body mass as well as the significantly lower BMI values in female ballet dancers, as compared to the non-dancers. Ku et al. [40] revealed that BMI had an impact on postural control during the unipedal stance. However, it would be very difficult to find a control group of healthy women with BMIs matching those of professional ballet dancers. Hamilton et al. [41] stated, that significant anatomic differences separate elite dancers of both genders from the normal population.

Other limitation of our study can be that lower-extremity muscle strength, which can significantly affect balance level [42], was not evaluated. Giboin et al. [43] state, that the learning of dynamic balance tasks may depend on the lower limb power. According to Ambegaonkar et al. [44], subjects with greater hip flexor, extensor, and abductor strength had better balance scores.

The small sample size of the ballet dancers may be another limitation. The conclusions reached using a larger sample size would have a greater validity; however, the sample size in the present study is comparable to other similar studies. The group of ballet dancers in the current study comprised all the ballet dancers who were employed at the two professional ballet companies and passed the inclusion criteria. The two ballet companies (the National Theatre in Brno and the Moravian Theatre in Olomouc) rank among four largest in the Czech Republic.

## Conclusions

The results from the present study do not support the hypothesis that professional ballet dancers have a better general balance ability than untrained subjects and that they would perform better particularly under conditions with high demands on postural control. The ballet dancers achieved significantly better results only in standing after 10 whole-body turns (vestibular manipulation) but the explanation for this can be that ballet dancers possess a task-specific skill that allows them to perform better under this rather specific condition.

## Supporting information

S1 Data Data set. (XLSX)

## References

[1] Koutedakis Y, Sharp NCC. The fit and healthy dancer. Hoboken, NJ: Wiley; 1999.

[2] Bronner S. Differences in segmental coordination and postural control in a multi-joint dance movement: Développé arabesque. J Dance Med Sci. 2012;16(1): 26–35. 22390951

[3] Kimmerle M. Lateral bias, functional asymmetry, dance training and dance injuries. J Dance Med Sci. 2010;14(2): 58–66. 20507722

[4] Costa MSdS, Ferreira AdS, Felicio LR. Static and dynamic balance in ballet dancers: A literature review. Fisioter Pesqui. 2013;20(3): 299–305. 10.1590/S1809-29502013000300016

[5] Gerbino PG, Griffin ED, Zurakowski D. Comparison of standing balance between female collegiate dancers and soccer players. Gait Posture. 2007;26(4): 501–507. 10.1016/j.gaitpost.2006.11.205 17197186

[6] Crotts D, Thompson B, Nahom M, Ryan S, Newton RA. Balance abilities of professional dancers on select balance tests. J Orthop Sports Phys Ther. 1996;23(1): 12–17. 10.2519/jospt.1996.23.1.12 8749745

[7] Giboin L-S, Gruber M, Kramer A. Task-specificity of balance training. Hum Mov Sci. 2015;44: 22–31. 10.1016/j.humov.2015.08.012 26298214

[8] Hugel F, Cadopi M, Kohler F, Perrin P. Postural control of ballet dancers: A specific use of visual input for artistic purposes. Int J Sports Med. 1999;20(2): 86–92. 10.1055/s-2007-971098 10190767

[9] Golomer E, Crémieux J, Dupui P, Isableu B, Ohlmann T. Visual contribution to self-induced body sway frequencies and visual perception of male professional dancers. Neurosci Lett. 1999;267(3): 189–192. 10.1016/s0304-3940(99)00356-0 10381008

[10] de Mello MC, de Sá Ferreira A, Ramiro Felicio L. Postural control during different unipodal positions in professional ballet dancers. J Dance Med Sci. 2017;21(4): 151–155. 10.12678/1089-313X.21.4.151 29166985

[11] Michalska J, Kamieniarz A, Fredyk A, Bacik B, Juras G, Słomka KJ. Effect of expertise in ballet dance on static and functional balance. Gait Posture. 2018;64: 68–74. 10.1016/j.gaitpost.2018.05.034 29879630

[12] Simmons RW. Neuromuscular responses of trained ballet dancers to postural perturbations. Int J Neurosci. 2005;115(8): 1193–1203. 10.1080/00207450590914572 16040361

[13] Borg FG, Laxåback G. Entropy of balance—some recent results. J Neuroeng Rehabil. 2010;7(1): 38 10.1186/1743-0003-7-38 20670457 PMC2923165

[14] Riemann BL, Myers JB, Lephart SM. Comparison of the ankle, knee, hip, and trunk corrective action shown during single-leg stance on firm, foam, and multiaxial surfaces. Arch Phys Med Rehabil. 2003;84(1): 90–95. 10.1053/apmr.2003.50004 12589627

[15] Costa M, Peng CK, Goldberger AL, Hausdorff JM. Multiscale entropy analysis of human gait dynamics. PHYSICA A. 2003;330(1–2): 53–60. 10.1016/j.physa.2003.08.022 PMC9070539 35518362

[16] Costa M, Goldberger AL, Peng CK. Multiscale entropy analysis of complex physiologic time series. Phys Rev Lett. 2002;89(6): 068102 10.1103/PhysRevLett.89.068102 12190613

[17] Costa M, Goldberger AL, Peng CK. Multiscale entropy analysis of biological signals. Phys Rev E. 2005;71(2): 021906 10.1103/PhysRevE.71.021906 15783351

[18] Goldberger AL, Amaral LA, Glass L, Hausdorff JM, Ivanov PC, Mark RG, et al PhysioBank, PhysioToolkit, and PhysioNet: components of a new research resource for complex physiologic signals. Circulation. 2000;101(23): E215–E220. 10.1161/01.cir.101.23.e215 10851218

[19] Stins JF, Michielsen ME, Roerdink M, Beek PJ. Sway regularity reflects attentional involvement in postural control: Effects of expertise, vision and cognition. Gait Posture. 2009;30(1): 106–109. 10.1016/j.gaitpost.2009.04.001 19411174

[20] Roerdink M, Hlavackova P, Vuillerme N. Center-of-pressure regularity as a marker for attentional investment in postural control: A comparison between sitting and standing postures. Hum Mov Sci. 2011;30(2): 203–212. 10.1016/j.humov.2010.04.005 20542347

[21] Donker SF, Roerdink M, Greven AJ, Beek PJ. Regularity of center-of-pressure trajectories depends on the amount of attention invested in postural control. Exp Brain Res. 2007;181(1): 1–11. 10.1007/s00221-007-0905-4 17401553 PMC1914290

[22] Lobo da Costa PH, Azevedo Nora FGS, Vieira MF, Bosch K, Rosenbaum D. Single leg balancing in ballet: Effects of shoe conditions and poses. Gait Posture. 2013;37(3): 419–423. 10.1016/j.gaitpost.2012.08.015 22989743

[23] Schmitt H, Kuni B, Sabo D. Influence of professional dance training on peak torque and proprioception at the ankle. Clin J Sport Med. 2005;15(5): 331–339. 10.1097/01.jsm.0000181437.41268.56 16162992

[24] Lin CF, Lee IJ, Liao JH, Wu HW, Su FC. Comparison of postural stability between injured and uninjured ballet dancers. Am J Sports Med. 2011;39(6): 1324–1331. 10.1177/0363546510393943 21335350

[25] Perrin P, Deviterne D, Hugel F, Perrot C. Judo, better than dance, develops sensorimotor adaptabilities involved in balance control. Gait Posture. 2002;15(2): 187–194. 10.1016/S0966-6362(01)00149-7 11869913

[26] Maki BE, Holliday PJ, Fernie GR. Aging and postural control. A comparison of spontaneous- and induced-sway balance tests. J Am Geriatr Soc. 1990;38(1): 1–9. 10.1111/j.1532-5415.1990.tb01588.x 2295764

[27] Wolfson L, Whipple R, Derby CA, Amerman P, Nashner L. Gender differences in the balance of healthy elderly as demonstrated by dynamic posturography. J Gerontol. 1994;49(4): M160–M167. 10.1093/geronj/49.4.m160 8014390

[28] Shumway-Cook A, Woollacott MH. Motor control: Translating research into clinical practice. 4th ed Philadelphia, PA: Lippincott Williams & Wilkins; 2011 656 p.

[29] Rosengren KS, Rajendran K, Contakos J, Chuang LL, Peterson M, Doyle R, et al Changing control strategies during standard assessment using computerized dynamic posturography with older women. Gait Posture. 2007;25(2): 215–221. 10.1016/j.gaitpost.2006.03.009 16650765

[30] Vuillerme N, Burdet C, Isableu B, Demetz S. The magnitude of the effect of calf muscles fatigue on postural control during bipedal quiet standing with vision depends on the eye-visual target distance. Gait Posture. 2006;24(2): 169–172. 10.1016/j.gaitpost.2005.07.011 16226030

[31] Kiefer AW, Riley MA, Shockley K, Sitton CA, Hewett TE, Cummins-Sebree S, et al Lower-limb proprioceptive awareness in professional ballet dancers. J Dance Med Sci. 2013;17(3): 126–132. 10.12678/1089-313X.17.3.126 24069947

[32] Guillou E, Dupui P, Golomer E. Dynamic balance sensory motor control and symmetrical or asymmetrical equilibrium training. Clin Neurophysiol. 2007;118(2): 317–324. 10.1016/j.clinph.2006.10.001 17140847

[33] Hopper DM, Grisbrook TL, Newnham PJ, Edwards DJ. The effects of vestibular stimulation and fatigue on postural control in classical ballet dancers. J Dance Med Sci. 2014;18(2): 67–73. 10.12678/1089-313X.18.2.67 24844423

[34] Golomer E, Bouillette A, Mertz C, Keller J. Effects of mental imagery styles on shoulder and hip rotations during preparation of pirouettes. J Motor Behav. 2008;40(4): 281–290. 10.3200/JMBR.40.4.281-290 18628105

[35] Lin CW, Chen SJ, Su FC, Wu HW, Lin CF. Differences of ballet turns (Pirouette) performance between experienced and novice ballet dancers. Res Q Exerc Sport. 2014;85(3): 330–340. 10.1080/02701367.2014.930088 25141086

[36] Osterhammel P, Terkildsen K, Zilstorff K. Vestibular habituation in ballet dancers. Acta Otolaryngol. 1968;66(1–6): 221–228. 10.3109/00016486809126289 5304145

[37] Ringhof S, Stein T. Biomechanical assessment of dynamic balance: Specificity of different balance tests. Hum Mov Sci. 2018;58: 140–147. 10.1016/j.humov.2018.02.004 29438911

[38] Casabona A, Leonardi G, Aimola E, La Grua G, Polizzi CM, Cioni M, et al Specificity of foot configuration during bipedal stance in ballet dancers. Gait Posture. 2016;46: 91–97. 10.1016/j.gaitpost.2016.02.019 27131184

[39] Kiefer AW, Riley MA, Shockley K, Sitton CA, Hewett TE, Cummins-Sebree S, et al Multi-segmental postural coordination in professional ballet dancers. Gait Posture. 2011;34(1): 76–80. 10.1016/j.gaitpost.2011.03.016 21530267

[40] Ku PX, Abu Osman NA, Yusof A, Wan Abas WAB. Biomechanical evaluation of the relationship between postural control and body mass index. J Biomech. 2012;45(9): 1638–1642. 10.1016/j.jbiomech.2012.03.029 22507349

[41] Hamilton WG, Hamilton LH, Marshall P, Molnar M. A profile of the musculoskeletal characteristics of elite professional ballet dancers. Am J Sports Med. 1992;20(3): 267–273. 10.1177/036354659202000306 1636856

[42] Paillard T. Relationship between muscle function, muscle typology and postural performance according to different postural conditions in young and older adults. Front Physiol. 2017;8(585): 585 10.3389/fphys.2017.00585 28861000 PMC5559497

[43] Giboin LS, Gruber M, Kramer A. Motor learning of a dynamic balance task: Influence of lower limb power and prior balance practice. J Sci Med Sport. 2019;22(1): 101–105. 10.1016/j.jsams.2018.05.029 29921504

[44] Ambegaonkar JP, Mettinger LM, Caswell SV, Burtt A, Cortes N. Relationships between core endurance, hip strength, and balance in collegiate female athletes. Int J Sports Phys Ther. 2014;9(5): 604–616. 25328823 PMC4196325

